# EXPRESSION OF CONCERN: ‘Tripterine up-regulates miR-223 to alleviate lipopolysaccharides-induced damage in murine chondrogenic ATDC5 cells’

**DOI:** 10.1177/20587384211000845

**Published:** 2021-02-28

The Journal Editors and SAGE Publishing hereby issue an expression of concern for the following article:

Xuefu Li, Wei Wei, Zhongquan Zhao, Shuzhen Lv (2019) Tripterine up-regulates miR-223 to alleviate lipopolysaccharide-induced damage in murine chondrogenic ATDC5 cells. International Journal of Immunopathology and Pharmacology 33: 1–11. DOI: 10.1177/2058738418824521.

It has been brought to SAGE’s attention that this article may have been written (and possibly submitted) by a third-party agency, and that the contents of the article may be partially or wholly fabricated. SAGE is currently conducting a thorough investigation into this to establish the most appropriate way forward.

The Editor and SAGE strive to uphold the very highest standards of publication ethics and are committed to supporting the high standards of integrity of *International Journal of Immunopathology and Pharmacology*. Authors, reviewers, editors and interested readers should consult the ethics section of the SAGE website and the Committee on Publication Ethics (COPE) website for guidelines on publication ethics. *International Journal of Immunopathology and Pharmacology* is a member of the Committee on Publication Ethics (COPE).

